# Supplementary material for: Malic enzyme 1 knockout has no deleterious phenotype and is favored in the male germline under standard laboratory conditions
Source: PLoS One. 2024 Jun 6;19(6):e0303577. doi: 10.1371/journal.pone.0303577 (PMC11156412; doi:10.1371/journal.pone.0303577)
Supplement: S1 Table — Me1 KO mice were aged to adulthood and were euthanized after the 100-week timepoint. Heterozygous mice for the Me1 null allele were similarly aged to adulthood, but some were euthanized before the 100-week timepoint due to necessary consolidation measures in 2020. (DOCX) [file pone.0303577.s001.docx]

| **Mouse ID** | **Genotype** | **Sex** | **Age at Euthanization (weeks)** |
| --- | --- | --- | --- |
| 20 | Me1^−/−^ | Female | 124 |
| 23 | Me1^−/−^ | Female | 100 |
| 33 | Me1^−/−^ | Male | 100 |
| 40 | Me1^−/−^ | Male | 103 |
| 41 | Me1^−/−^ | Male | 103 |
| 42 | Me1^−/−^ | Female | 129 |
| 44 | Me1^−/−^ | Female | 106 |
| 46 | Me1^−/−^ | Female | 103 |
| 22 | Me1^+/−^ | Female | 98 |
| 32 | Me1^+/−^ | Male | 80 |
| 30.1 | Me1^+/−^ | Female | 124 |
| 30.2 | Me1^+/−^ | Female | 124 |
