# Supplementary figures and images for: Malic enzyme 1 knockout has no deleterious phenotype and is favored in the male germline under standard laboratory conditions
Source: PLoS One. 2024 Jun 6;19(6):e0303577. doi: 10.1371/journal.pone.0303577 (PMC11156412; doi:10.1371/journal.pone.0303577)

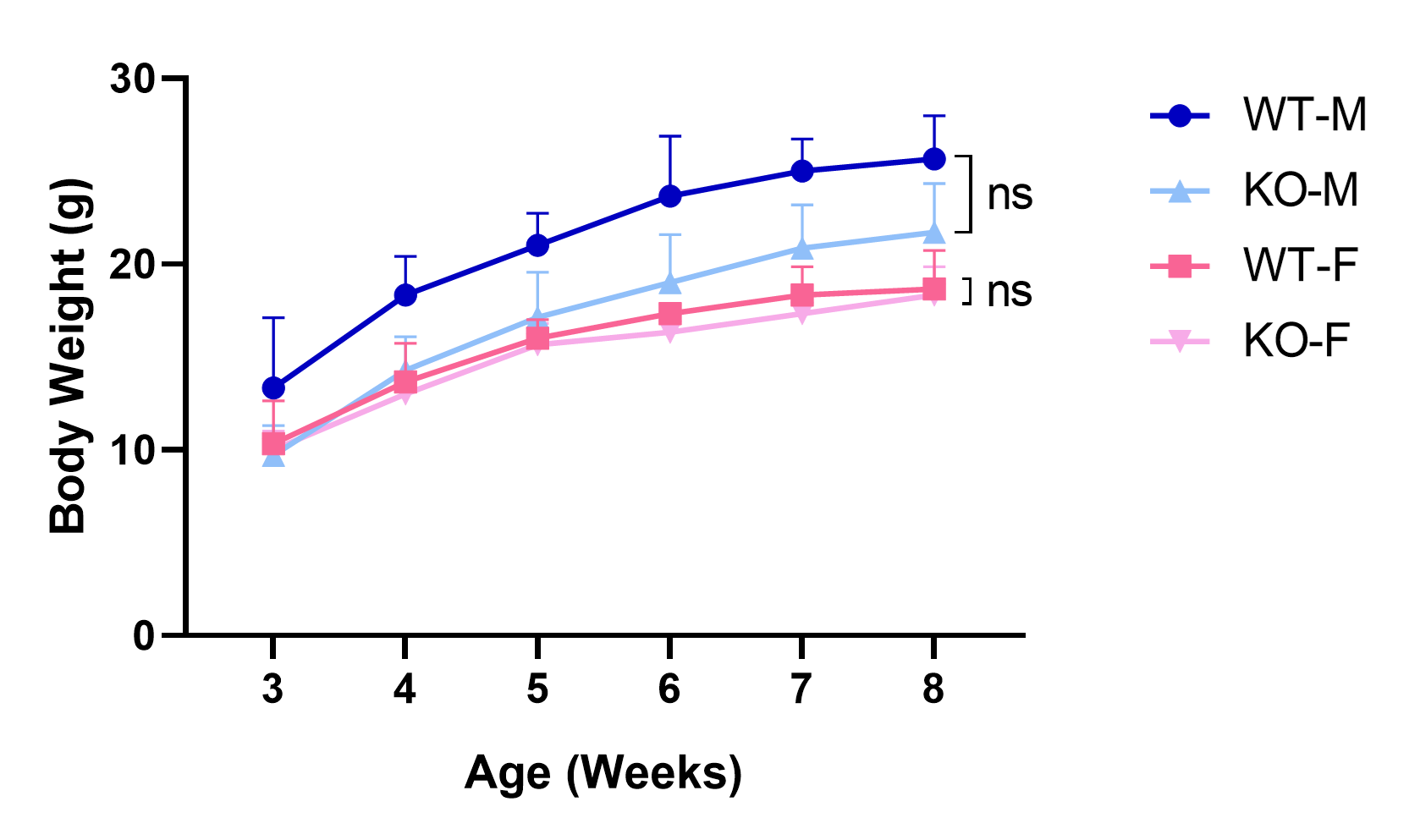

Supplement: S1 Fig — Body weights of wild-type male (WT-M; n = 3) and female (WT-F; n = 3) mice were tracked Me1 KO male (KO-M; n = 7) and female (KO-F; n = 3) mice weekly from 3–8 weeks of age. An initial discrepancy in body weight at 3 weeks of age between the WT-M and KO-M mice was noted specifically in this cohort, but body weight gain and growth rates did not reach significant differences at any time point. (TIF) [file pone.0303577.s003.tif]

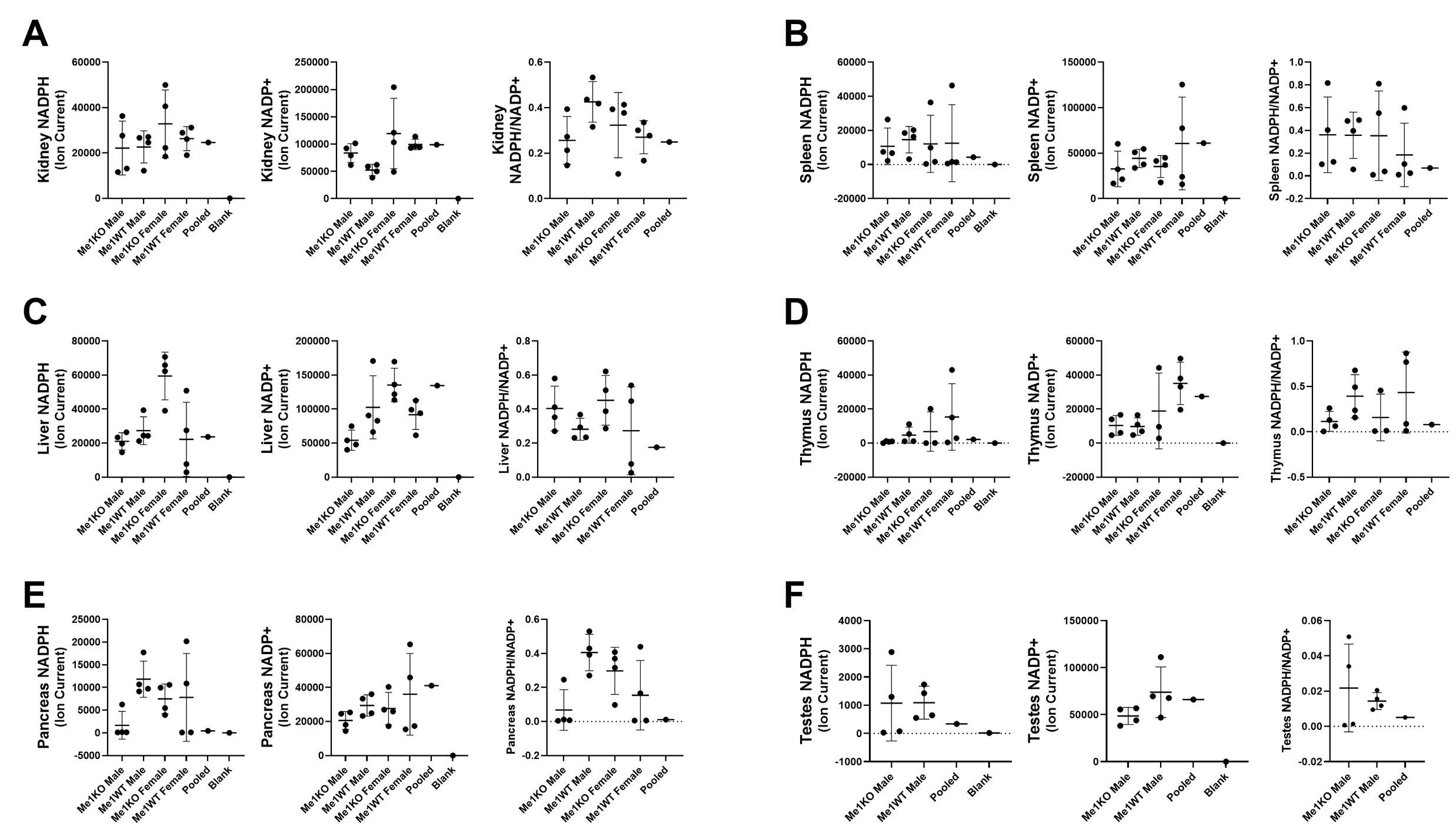

Supplement: S2 Fig — NADP+, NADPH, and NADP+/NADPH ratios in (A) liver, (B) thymus, (C) testes, (D) kidney, (E) pancreas, and (F) spleen from Me1 wild type versus Me1 null animals, as assessed by LC-MS and presented by sex. Pooled samples were included as controls; blanks assessed instrument performance and represent background without biological sample. (TIF) [file pone.0303577.s004.tif]

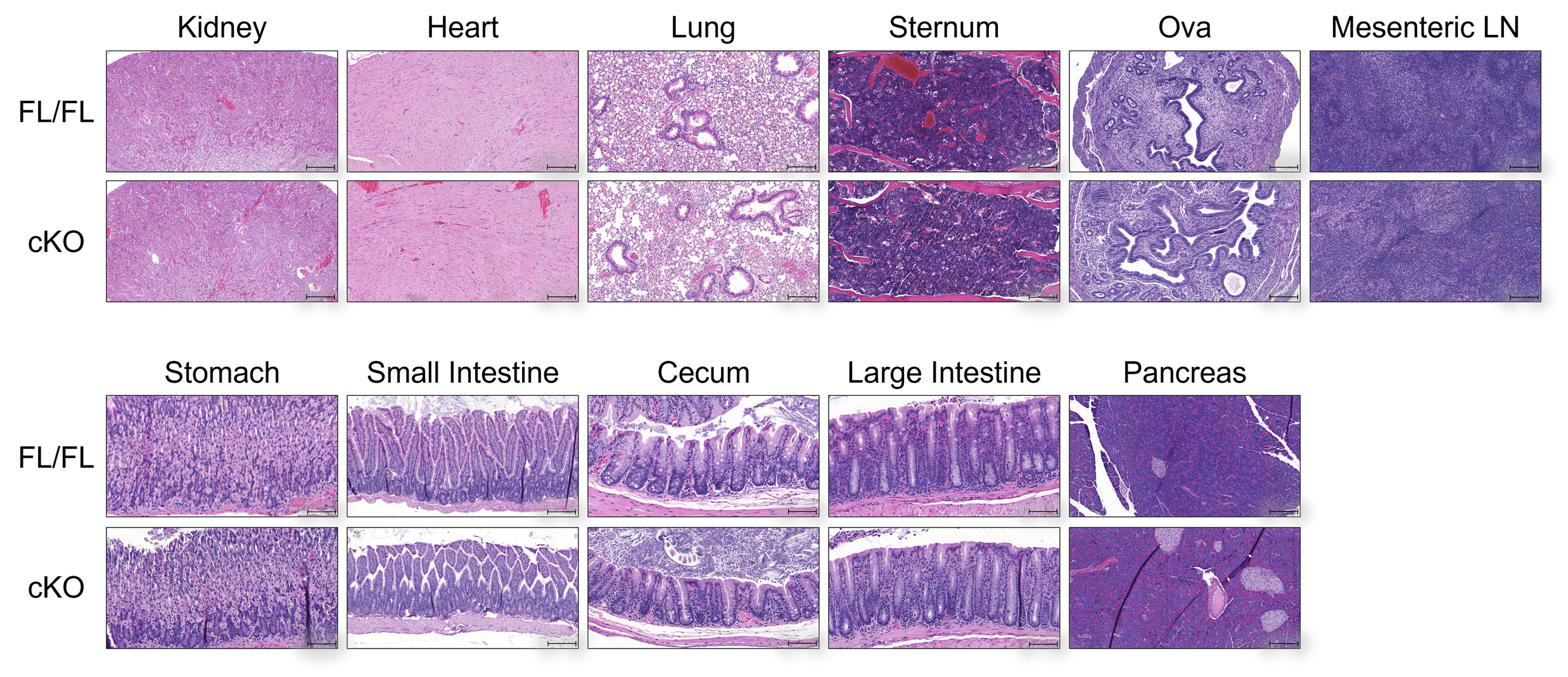

Supplement: S3 Fig — Kidneys imaged at 5X (scale bar = 400 μm). Stomach, cecum, and large intestine imaged at 20X (scale bar = 100 μm). All other tissues imaged at 10X (scale bar = 200 μm). (TIF) [file pone.0303577.s005.tif]

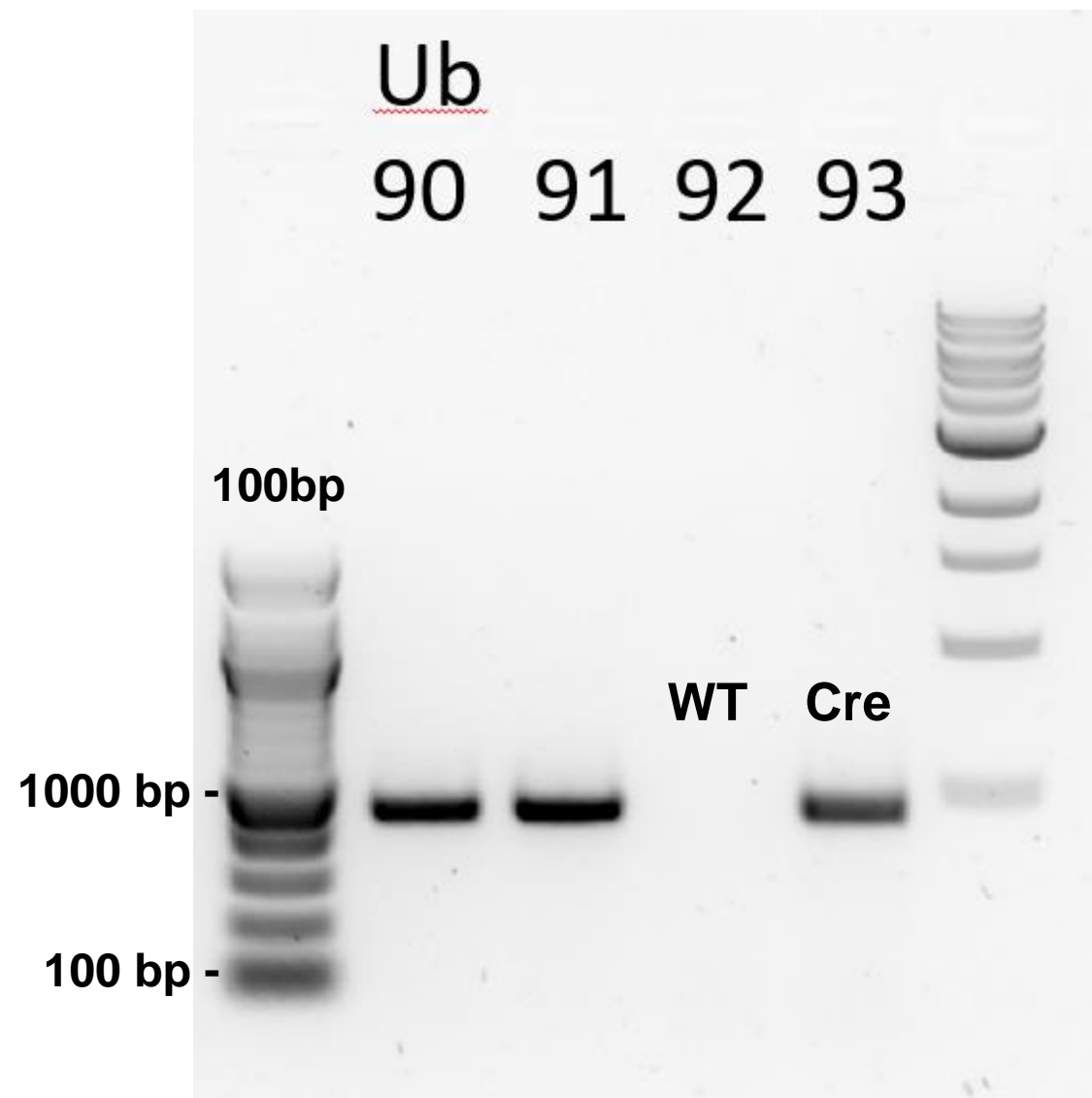

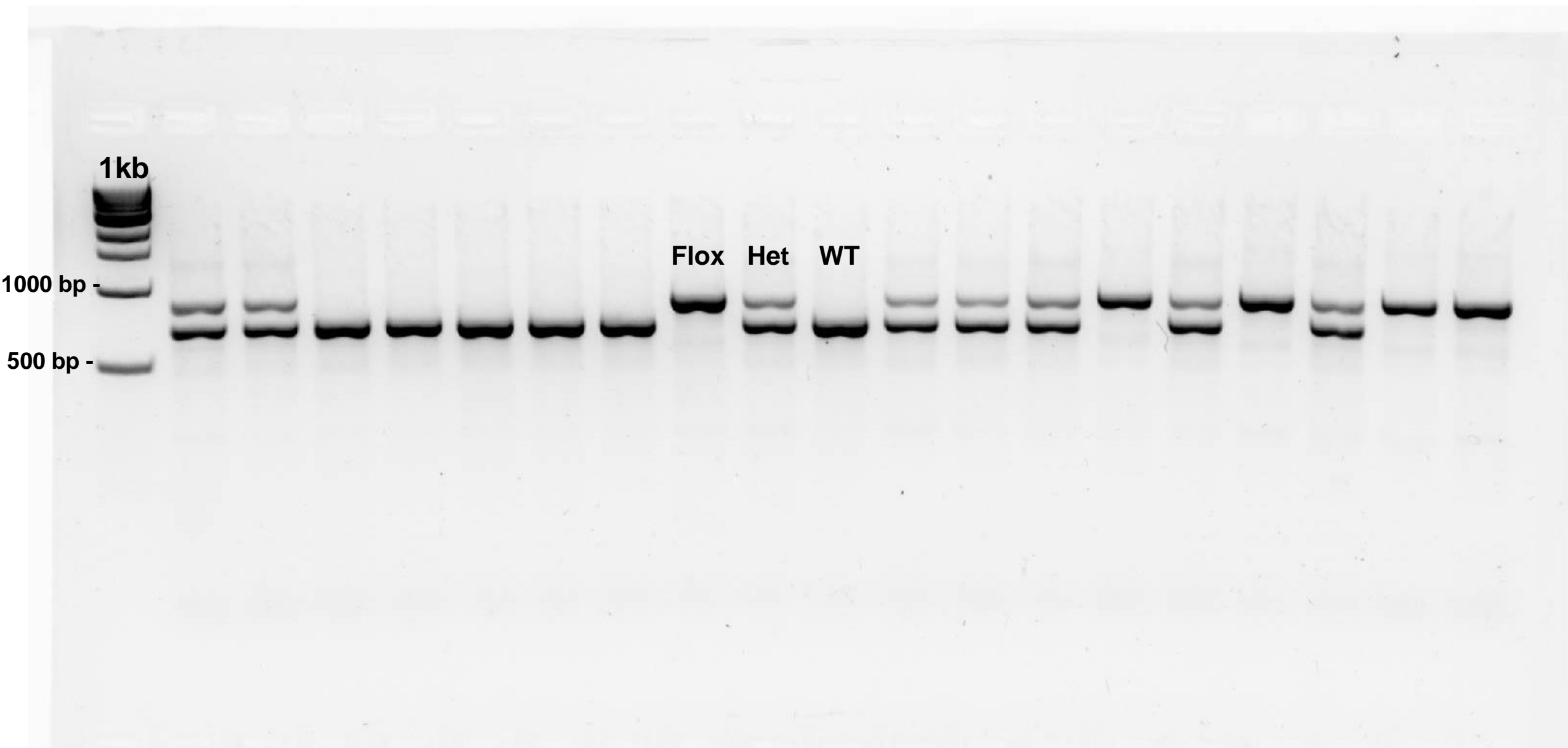

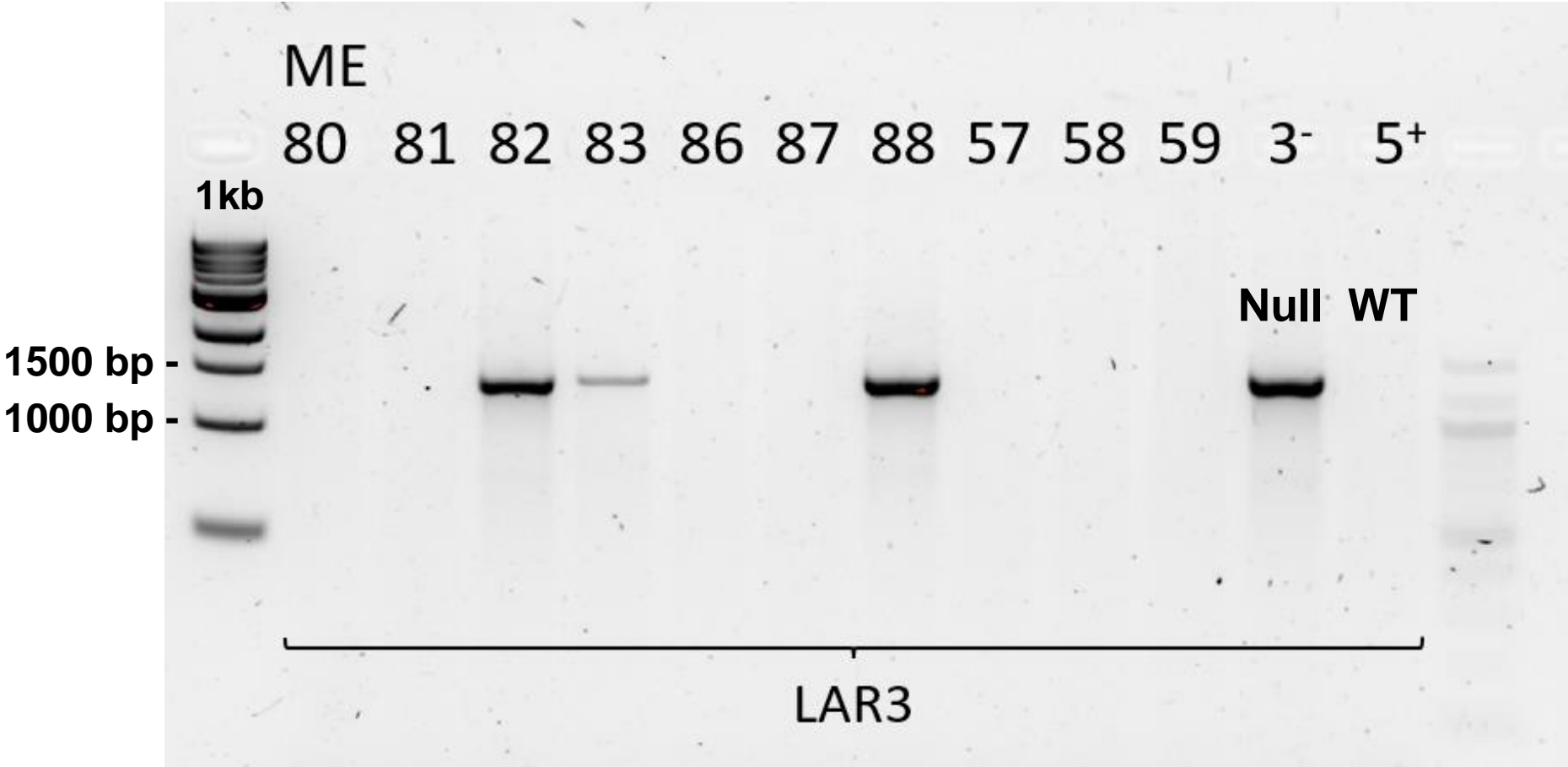

Supplement: S4 Fig — Uncropped gel images from Fig 1D–1F. (PDF) [file pone.0303577.s006.pdf]
