# Supplementary material for: Malic enzyme 1 knockout has no deleterious phenotype and is favored in the male germline under standard laboratory conditions
Source: PLoS One. 2024 Jun 6;19(6):e0303577. doi: 10.1371/journal.pone.0303577 (PMC11156412; doi:10.1371/journal.pone.0303577)
Supplement: Attachment — Submitted filename: Alektiar, Shan, et al. Rebuttal Letter_final.docx [file pone.0303577.s007.docx]

Rebuttal of Alektiar and Shan, et al.
Manuscript Number: PONE-D-23-20509

**We are truly grateful for the time and effort put forth by the Editors and Reviewers at *PLOS ONE*. We have addressed the experimental and textual concerns raised, and we believe that the helpful comments have allowed us to strengthen the conclusions presented in the accompanying manuscript. Below is a point-to-point response to these comments; referee remarks are presented in plain text, our responses in bold.**

**REVIEWERS’ COMMENTS**

**Reviewer 1**

In the manuscript by Alektiar JM and Shan M, titled “Malic enzyme 1 knockout has no deleterious phenotype and is favored in the male germline under standard laboratory conditions”, the authors describe the interrogation of loss of malic enzyme 1 (ME1) using in vivo genetic models. The authors demonstrate that loss of ME1, either constitutively deleted during animal development or deleted in adult animals, had no detrimental effects on animals. Interestingly, they show that the ME1 KO genotype is favored when maternally transmitted to male progeny. This is a clear and thorough study; only minor suggestions are provided to improve the study.

**Response: We thank the reviewer for the supportive comments and suggestions. We have incorporated this feedback, and it has served well to provide clarity and further strengthen our conclusions.**

Minor Suggestions

1. The authors do not measure NADPH/NADP+ or triglyceride levels in the same tissues where they observe ME1 genetic/protein deletion. Measuring NADPH/NADP+ and triglycerides would significantly add to the conclusions, especially since the authors discuss how ME1 is reported to promote NADPH generation and triglyceride production. Regardless of the outcome, this data would be very informative. If these metrics don’t change, it could suggest an explanation for the dispensability of ME1. If these metrics do change, it could potentially suggest that these changes are not sufficient to impair animal development and homeostasis.

**Response: This is an excellent suggestion, and we agree with the referee’s interpretations. Indeed, upon analyzing NADPH/NADP+ ratios in *Me1* null animals versus wild type, we did not observe significant differences across six tissues, collectively or when separated by sex (Figure 3D-I, Supplementary Figure 3). Our interpretation of this data, like that of the referee’s, is that cells/organs/animals adapt to ME1 loss under routine conditions by either upregulating other NADPH-generating pathways or only requiring ME1-mediated NADPH under special circumstances not evident in this study.**

2. The authors could improve the clarity of Figure 1 by adding details to Fig. 1D, 1E, and 1F to show, along with the mouse strains, what the PCR experiment is amplifying. For example, having “Me1 null lacZ reporter” in 1D and “loxP” in Figure 1E. Alternatively, the authors could include the description of the primer pairs used in Fig. 1D-1F in the targeting strategy schematics in Fig. 1A-1C.

**Response: We have updated Figure 1 to include the primer binding sites.**

3. It is unclear why the loss of protein expression is described as a “knockdown” of ME1 throughout the manuscript instead of “knockout”, which might be more appropriate.

**Response: Knockdown was used to refer to results from the conditional knockout animal, since knockout was not complete on a tissue level. However, the referee’s point is well taken, and knockdown has been replaced with knockout throughout the manuscript.**

Reviewer #2: The present manuscript describes 2 mouse models with lack of ME1. These models are used to validate that ME1 is not essential for normal development or physiological function, at least in the absence of stresses like infection or starvation. The data are objectively presented and compelling. There is also the surprising and intriguing observation that eggs lacking ME1 are preferentially transmitted, relative to WT, at least in the case of male offspring. This could possibly relate to an alternative NADPH pathway, G6PD, being X-linked, although why this would favor ME1 loss is unclear. Overall, this is a clear and valuable contribution and the mouse models will be valuable going forward for the community.

**Response: We genuinely appreciate the thoughtful feedback and recognition of the implications of this work. We also want to thank the referee for supporting this work in its initial format, without the need for additional experimentation.**
